# Clinical audit of the inclusion of the Lester Tool details in discharge documents at Foss Park Hospital, York

**DOI:** 10.1192/bjo.2021.265

**Published:** 2021-06-18

**Authors:** Kayleigh Jones, Shona McIlrae, Karen Ball, Rohma Tahir

**Affiliations:** Tees Esk and Wear Valley NHS Foundation Trust

## Abstract

**Aims:**

Patients with serious mental health illnesses die on average 15–20 years before the rest of the general population. Anti-psychotic medication, lifestyle and difficulty accessing healthcare services all have a detrimental effect on their life expectancy. To improve outcomes for these patients the Lester Tool; a method to assess the cardiovascular health of patients and implement change, was developed. Including the Lester Tool information in discharge letters allows transfer of information to other care providers (mainly GP's) who can implement and monitor any interventions made, improving outcomes for our patients. With this in mind, discharge documents should contain all of the information listed in the Lester Tool.

We aimed to check if 100% of data required by the Lester Tool is included in discharge documents of the inpatients at Foss Park Hospital.

**Method:**

20 patients from each of the male and female wards at Foss Park hospital, discharged in September or October 2020, were identified. A review of the discharge documents established whether the smoking status, BMI, ECG, blood pressure and blood results of each patient were recorded.

**Result:**

Of the 40 discharges, none had 100% compliance. On average across both wards; only 23% of the Lester tool information was included in the documents. On the female ward, 40% had none of data recorded, while on the male ward, 15% had none of the data recorded. Across both wards, not a single patient had details about their cholesterol ratio recorded, only 50% of BMI's were recorded and only 27% had a smoking status included.

**Conclusion:**

Our results have shown that compliance with the Lester Tool falls short of what is expected. As a result, information about the physical health of our patients is not being communicated effectively with other care providers. This in turn can prevent patients being offered interventions needed to improve their cardiovascular health.

Identifying this shortcoming in the transfer of information will allow us to educate the staff in our organisation and ensure that all the necessary physical health details will be included in future discharge documents. The result being improved outcomes and longer life expectancy of patients with serious mental illnesses, satisfying the purpose of the Lester Tool.

